# Enhanced Thermoelectric Performance of SnTe via Introducing Resonant Levels

**DOI:** 10.3390/molecules29204974

**Published:** 2024-10-21

**Authors:** Manman Yang, Jin Jia, Haijun Yu, Yimin Li, Lu Han, Hairui Sun, Haowen Jia, Yuanyuan Zhu

**Affiliations:** 1 School of Electronic Engineering, Huainan Normal University, Huainan 232038, China; ymm@hnnu.edu.cn (M.Y.); haijun20030@163.com (H.Y.); lymhnnu@163.com (Y.L.); hanlu@hnnu.edu.cn (L.H.); 2 Key Laboratory of Spin Electron and Nanomaterials of Anhui Higher Education Institutes, Suzhou University, Suzhou 234000, China; jiahaowen100823@163.com; 3 Laboratory of High Pressure Physics and Material Science, School of Physics and Physical Engineering, Qufu Normal University, Qufu 273165, China; hairuisun1216@qfnu.edu.cn

**Keywords:** thermoelectrics, tin telluride, high pressure and high temperature, In doping

## Abstract

SnTe has emerged as a non-toxic and environmentally friendly alternative to the high-performance thermoelectric material PbTe, attracting significant interest in sustainable energy applications. In our previous work, we successfully synthesized high-quality SnTe with reduced thermal conductivity under high-pressure conditions. Building on this, in this work, we introduced indium (In) doping to further decrease thermal conductivity under high pressure. By incorporating resonance doping into the SnTe matrix, we aimed to enhance the electrical transport properties while maintaining low thermal conductivity. This approach enhances the Seebeck coefficient to an impressive 153 μVK^−1^ at 735 K, marking a notable enhancement compared to undoped SnTe. Furthermore, we noted a substantial decrease in total thermal conductivity, dropping from 6.91 to 3.88 Wm^−1^K^−1^ at 325 K, primarily due to the reduction in electrical conductivity. The synergistic impact of decreased thermal conductivity and heightened Seebeck coefficient resulted in a notable improvement in the thermoelectric figure of merit (*ZT*) and average *ZT*, achieving approximately 0.5 and 0.22 in the doped samples, respectively. These advancements establish Sn_1−*x*_In*_x_*Te as a promising candidate to replace PbTe in thermoelectric applications, providing a safer and more environmentally sustainable option.

## 1. Introduction

Thermoelectric materials hold great promise for the advancement of energy-conversion technologies, as they enable the direct and efficient transformation of waste heat into usable electrical power, without the need for noisy and environmentally harmful mechanical components such as turbines or generators [1,2,3,4,5]. This unique capability has sparked significant interest in the scientific community, with numerous studies exploring the potential applications of thermoelectric materials in a wide range of fields, from renewable energy to aerospace [6,7,8]. A key parameter in assessing this efficiency is the dimensionless figure of merit, *ZT*, defined by the equation *ZT* = (*S*^2^*σ*/*κ*)*T*, where *S* is the Seebeck coefficient, *σ* is electrical conductivity, *κ* is total thermal conductivity, and *T* is absolute temperature [9,10,11]. Both *S* and *σ* are highly dependent on carrier concentration, which can be precisely controlled through a strategic doping technique [12,13,14]. Certainly, it is one of the important means of improving the thermoelectric properties of thermoelectric material. The total thermal conductivity, *κ*, comprises electronic thermal conductivity (*κ*_ele_) and phonon thermal conductivity (*κ*_ph_), expressed as *κ = κ*_ele_ + *κ*_ph_ [15,16]. *κ*_ele_ is influenced by the movement of charge carriers, while *κ*_ph_ is associated with the vibrations of the material’s atomic lattice. *κ*_ph_ often exhibits a weaker dependence on other material parameters, making it a prime target for enhancement strategies [17,18]. Researchers typically focus on reducing *κ*_ph_ through various approaches to improve the *ZT* value, such as introducing lattice defects to scatter phonons [19,20,21]. By achieving a higher *ZT* value, thermoelectric materials can operate with greater efficiency, paving the way for more practical and widespread applications in energy harvesting, refrigeration, and waste heat-recovery systems.

Despite the remarkable progress in the development of thermoelectric materials, lead telluride (PbTe) continues to dominate the landscape of power-generation technologies [22,23]. This dominance is primarily due to PbTe’s exceptional thermoelectric properties, which include a high Seebeck coefficient and low thermal conductivity. However, the inherent toxicity of Pb poses significant limitations to the widespread application of PbTe-based systems [24,25,26]. This toxicity poses environmental and health risks, making PbTe unsuitable for certain applications and restricting its use in certain geographical regions. In this context, tin telluride (SnTe) emerges as a promising substitute for PbTe, offering a non-toxic, environmentally friendly option by replacing toxic Pb with the more abundant and eco-friendly Sn [27]. Moreover, SnTe shares a similar crystal structure and analogous chemical and physical properties with PbTe, which facilitates the transition of existing PbTe thermoelectric technologies to SnTe-based systems [28], thereby reducing the cost and time associated with the development of new thermoelectric devices.

However, SnTe exhibits challenges such as a high concentration of Sn vacancies, leading to a reduced Seebeck coefficient and increased thermal conductivity [29]. Additionally, SnTe’s narrow band gap of approximately 0.18 eV and the substantial energy disparity (0.3–0.4 eV) between its light and heavy hole bands diminish the contribution of heavy holes to electronic transport, thereby compromising its thermoelectric performance relative to PbTe [30,31]. To address these challenges, doping with appropriate elements such as Mg [32], Mn [33], Hg [34], and Ca [35] has been proposed to optimize band structure and carrier concentration, enhancing the Seebeck coefficient and reducing thermal conductivity. In our previous studies, we found and confirmed that high pressure as an independent variable from temperature and composition can enhance the solubility limits and effectiveness of dopants, thereby tuning the band structure and improving thermoelectric performance without forming impurity phases [36,37]. Furthermore, high pressure can induce the formation of lattice defects, such as lattice distortion and dislocations, which can scatter phonons and reduce thermal conductivity.

In this study, we introduce an innovative and expedited fabrication process for high-quality SnTe compounds, leveraging the transformative potential of high-pressure techniques. This method not only ensures the structural integrity and compositional uniformity of the SnTe samples but also enables precise control over the material’s properties, which is crucial for enhancing its thermoelectric performance. Moreover, a pivotal aspect of our research involves the strategic doping of SnTe with indium (In) under high pressure. The introduction of In effectively modulates the Seebeck coefficient, particularly at room temperature, by creating resonant energy levels near the Fermi level in the valence band. This modification results in a notable enhancement of the power factor to approximately 1.6 mW m^−1^ K^−2^. Concurrently, the thermal conductivity is significantly reduced, achieving an exceptionally low value of approximately 2.16 W m^−1^ K^−1^ at 735 K in SnTe doped with 0.25 at.% In. The balance between an improved power factor and reduced thermal conductivity culminates in a figure of merit, *ZT*, of approximately 0.5 at 735 K. This investigation not only demonstrates the effectiveness of high-pressure fabrication and In doping in enhancing the thermoelectric properties of SnTe but also elucidates the underlying mechanisms responsible for these improvements.

## 2. Results and Discussion

**Figure 1:**
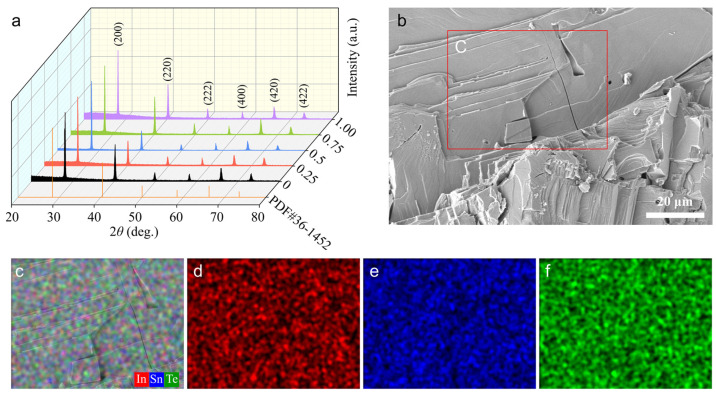
(**a**) Room temperature powder X-ray diffraction patterns for In_x_Sn_1−x_Te (x = 0, 0.25 at.%, 0.5 at.%, 0.75 at.%, 1 at.%). (**b**) SEM image for In_0.005_ Sn_0.995_Te; (**c**–**f**) the EDS elemental mapping of In, Sn, and Te, respectively.

Figure 1a meticulously displays the powder X-ray diffraction (XRD) patterns for In*_x_*Sn_1−*x*_Te samples with varying In concentrations (*x* = 0, 0.25 at.%, 0.5 at.%, 0.75 at.%, 1 at.%). These patterns are pivotal in characterizing the crystallographic structure of the synthesized samples. The well-defined diffraction peaks observed in the patterns are in excellent agreement with the NaCl-type crystal structure (space group Fm$\bar{3}$m, SnTe: PDF#36-1452; a = b = c = 6.328 Å) [32], indicating that the substitution of Sn with In does not alter the fundamental crystal structure. Importantly, no secondary phases detected within the resolution limits of the XRD apparatus suggest a high degree of phase purity in the samples, which is crucial for achieving optimal thermoelectric performance. In Figure 1b, an SEM image of the In_0.0025_Sn_0.9975_Te sample provides a detailed morphological view of the material’s surface. The image reveals a dense polycrystalline structure, indicative of the successful synthesis of the sample under the high-pressure conditions of 4.0 GPa. The fine-grained structure of the polycrystalline material is expected to facilitate enhanced phonon scattering due to the presence of numerous grain boundaries. This scattering is a key factor in reducing phonon thermal conductivity, which is essential for improving the *ZT* value. To further corroborate the composition and uniformity of the samples, EDS elemental mapping was performed. Figure 1c–f illustrates the EDS mapping results for the In_0.0025_Sn_0.9975_Te sample, revealing the distribution of In, Sn, and Te elements. The mapping data confirm the homogeneous distribution of these elements throughout the polycrystalline SnTe structure, which is vital for ensuring consistent material properties and reliable thermoelectric performance. The even distribution of In dopants is particularly significant, as it confirms the effectiveness of the doping process in modifying the material’s electronic and thermal transport properties. The combination of XRD, SEM, and EDS analyses provides a comprehensive understanding of the structural and compositional integrity of the In*_x_*Sn_1−*x*_Te samples, underpinning the subsequent thermoelectric performance evaluations.

In order to thoroughly evaluate the electrical transport properties of the In*_x_*Sn_1−*x*_Te (*x* = 0, 0.25 at.%, 0.5 at.%, 0.75 at.%, 1 at.%), a systematic investigation of the Hall coefficient (*R*_H_), carrier concentration (*n*_H_), and Hall mobility (*μ*_H_) was conducted at room temperature. The findings from these measurements are meticulously documented and summarized in Table 1, providing a comprehensive overview of the charge transport characteristics within the material system. The measurement of the *R*_H_ revealed consistently positive values across all samples, which is indicative of hole-dominated charge transport in In*_x_*Sn_1−*x*_Te. This observation is consistent with the material’s *p*-type semiconducting nature. A notable trend observed in the *n*_H_ as a function of In content is its non-monotonic variation. Initially, at approximately 0.25 at.% In doping, there is a decrease in *n*_H_. This can be explained by the high concentration of Sn vacancies in the undoped SnTe, which act as effective hole generators, thus increasing the hole concentration. As In is introduced into the lattice, it preferentially occupies these Sn vacancies. While In is indeed a *p*-type dopant, its contribution to hole generation is less effective than that of the Sn vacancies. Consequently, the *n*_H_ decreases as the In atoms begin to fill these vacancies. However, as the In doping concentration increases beyond the point of saturation for Sn vacancy filling, the excess In atoms start to replace Sn atoms within the lattice. This substitution leads to an increase in the *p*-type charge carrier concentration, which aligns with the observations reported in the literature [38]. This transition from vacancy filling to atom substitution is a pivotal factor in the material’s electronic behavior and its potential for thermoelectric applications. Concurrently, *μ*_H_ of the In-doped samples exhibits a significant decline from ~933 to 124 cm^2^ V^−1^ s^−1^ as the In content increases. This reduction in mobility can be attributed to a combination of factors, including the increased *n*_H_ and enhanced impurity scattering. The increased *n*_H_ leads to more frequent carrier–carrier interactions, which in turn impede the flow of charge and reduce mobility. Additionally, the presence of impurities introduced by the doping process increases scattering events, further hindering the carriers’ movement and contributing to the decreased mobility. The interplay between *n*_H_ and scattering mechanisms is a critical aspect of optimizing the thermoelectric performance of doped semiconductor materials.

**Figure 2:**
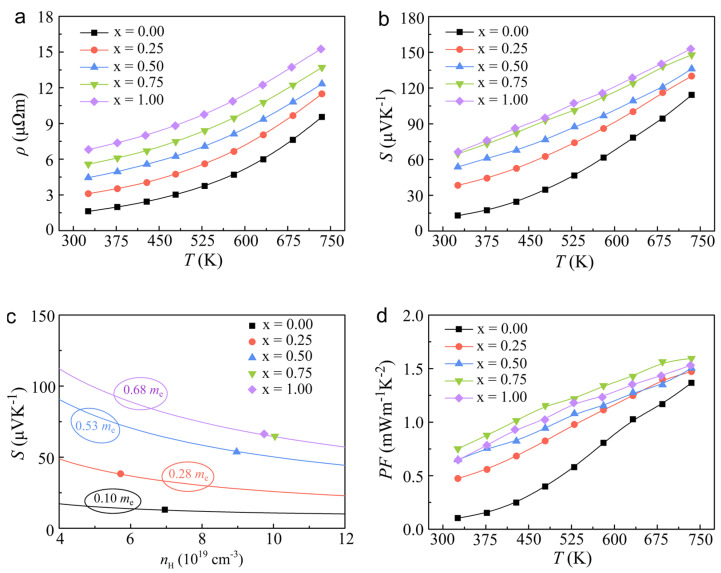
Temperature dependence of electrical transport properties: (**a**) Electrical resistivity (*ρ*); (**b**) Seebeck coefficient (*S*); (**c**) the relationship of Seebeck coefficient with carrier concentration; (**d**) power factor (*PF*) for In*_x_*Sn_1−*x*_Te (*x* = 0, 0.25 at.%, 0.5 at.%, 0.75 at.%, 1 at.%).

Figure 2 provides a detailed illustration of the temperature-dependent electrical transport properties of the In*_x_*Sn_1−*x*_Te samples, measured over a broad temperature range from 325 to 735 K. The data presented in this figure offer valuable insights into the behavior of these materials as thermoelectric converters. As shown in Figure 2a, the resistivity (*ρ*) of all the samples exhibits an upward trend with increasing temperature, which is characteristic of degenerate semiconductors. This behavior indicates that the charge carriers’ concentration is high enough to maintain a significant population of electrons in the conduction band even as the temperature rises, leading to increased scattering events that impede electrical conductivity. A notable observation is that the resistivity of the In-doped samples is consistently higher than that of the undoped SnTe throughout the entire temperature range. This increase in resistivity can be rationalized by considering the relationship *ρ* = 1/(*n*_H_e*μ*_H_), where *n*_H_ is the carrier concentration and *μ*_H_ is the Hall mobility. The data in Table 1 suggest that the reduction in *μ*_H_ has a more pronounced effect on *ρ* than the increase in *n*_H_. This is contrary to the typical expectation that an increase in *n*_H_ would lead to a decrease in resistivity. In a surprising turn, the Seebeck coefficient (*S*) of all samples increases with In doping, contrary to the common expectation that increased *n*_H_ would lead to a decrease in *S*. Specifically, at room temperature, the *S* increases dramatically from 13 to 66 μV K^−1^ with 1.0 at.% In doping, it reaches a peak value of 153 μV K^−1^ at 735 K. This corresponds to enhancements of ~408% and 34% for In_0.1_Sn_0.9_Te at 325 K and 735 K, respectively. This significant enhancement in *S* can be primarily attributed to the introduction of resonant energy levels in the valence band by In doping, which alters the electronic structure and enhances the effective mass of the charge carriers. To gain a deeper understanding of the electrical transport mechanism in the In*_x_*Sn_1−*x*_Te samples, we calculated the modified density of states (m*_DOS_) based on the single parabolic band (SPB) model [39], as presented in Figure 2c. The enhanced m*_DOS_ for the In-doped samples is a key factor contributing to the increased *S*, indicating substantial modifications to the band structure of SnTe following the doping process. The combination of a high *S* and a relatively low *ρ* results in an improved power factor (*PF*), as depicted in Figure 2d. The *PF* remains elevated across all samples from room temperature to elevated temperatures, indicating a robust thermoelectric performance. Notably, the *PF* for the *x* = 0.75 at.% sample reaches approximately 0.75 mW m^−1^ K^−2^ at 325 K, which is significantly higher than the value for undoped SnTe (~0.1 mW m^−1^ K^−2^). This superior *PF* correlates with enhanced output power [40], making the In-doped SnTe samples promising candidates for thermoelectric applications where high power output is required. The elevated average power factor (*PF*_avg_) over the measured temperature range underscores the potential of these materials for practical use in converting waste heat into electricity.

**Figure 3:**
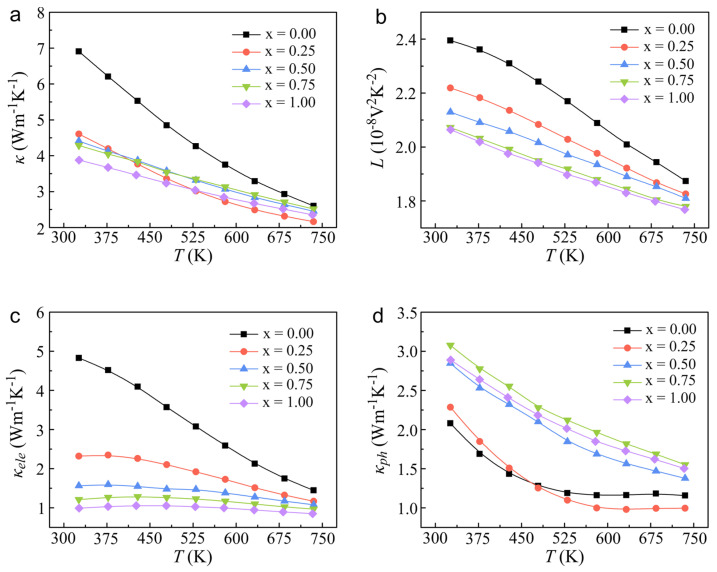
Temperature dependence of thermal transport properties: (**a**) Total thermal conductivity (*κ*); (**b**) Lorentz number (*L*); (**c**) electronic thermal conductivity (*κ*_ele_); and (**d**) phonon thermal conductivity (*κ*_ph_) for In*_x_*Sn_1−*x*_Te (*x* = 0, 0.25 at.%, 0.5 at.%, 0.75 at.%, 1 at.%).

Figure 3 delineates the temperature-dependent thermal transport properties of the In*_x_*Sn_1−*x*_Te samples, showcasing a notable trend of significant reductions in thermal conductivity (*κ*) with increasing temperature. This behavior is particularly noteworthy as it suggests that the material’s thermal conductivity is not influenced by bipolar transport effects, which are often observed in materials with high carrier concentrations. The incorporation of In into the SnTe lattice has a pronounced impact on the thermal conductivity across the temperature range of 325–735 K. For instance, the In_0.0025_Sn_0.9975_Te sample experiences thermal conductivity reductions of approximately 33% at 325 K and 17% at 735 K compared to the undoped SnTe. This result can be compared with our previous results of nanostructured SnTe synthesized by high-energy ball milling and hot compression techniques [41]. This substantial reduction in thermal conductivity is a significant advantage for thermoelectric materials, as it contributes to a higher thermoelectric figure of merit (*ZT*) and improved energy-conversion efficiency. To elucidate the mechanisms underlying this decrease in thermal conductivity, a comprehensive analysis of the contributions from electronic thermal conductivity (*κ*_ele_) and phonon thermal conductivity (*κ*_ph_) was conducted. As illustrated in Figure 3c, *κ*_ele_ was calculated using the Wiedemann–Franz law, expressed as *κ*_ele_ = *LσT*, where *L* represents the Lorenz constant. The Lorenz constant was determined using the formula *L* = 1.5 + exp(−|*S*|/116) [42], as depicted in Figure 3b. This method allows for the quantification of the electronic contribution to thermal conductivity, which is highly dependent on the carrier concentration and electrical conductivity. The *κ*_ph_ was derived by subtracting *κ*_ele_ from the *κ* [28,43], as shown in Figure 3d. The reduction in electrical conductivity due to doping with In diminishes the electronic contribution to thermal conductivity, leading to a decrease in *κ*. Additionally, the observed *κ*_ph_ values for In*_x_*Sn_1−*x*_Te in this study are lower than those previously reported for SnTe with nanostructured architectures [38], underscoring the effectiveness of the high pressure in modulating thermal transport properties. The high-pressure conditions during synthesis are known to enhance phonon scattering, which can effectively reduce phonon thermal conductivity [44]. The results presented in Figure 3 highlight the synergistic effects of In doping and high-pressure synthesis on the thermal conductivity of SnTe. These modifications, combined with the enhanced electrical transport properties observed in the previous sections, contribute to a significantly improved thermoelectric performance of the In*_x_*Sn_1−*x*_Te samples.

**Figure 4:**
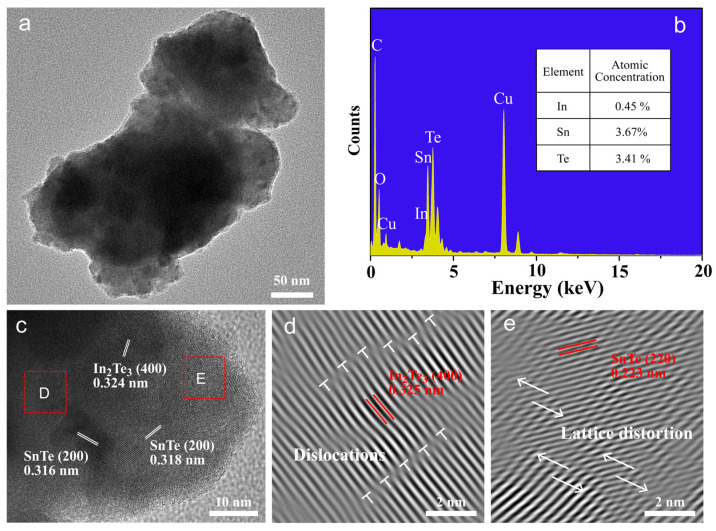
Microstructure and spectrum analysis of produced In_0.005_Sn_0.995_Te sample: (**a**) A low-magnification image; (**b**) the corresponding spectrum analysis; (**c**) the high-resolution TEM image; (**d**,**e**) IFFT images of the selected distortion area (area D and E defined by the red square) in (**c**).

The microstructural analysis of the synthesized In*_x_*Sn_1−*x*_Te samples was a critical step in understanding the phonon scattering mechanisms that underpin their thermoelectric properties. Low-magnification TEM images, presented in Figure 4a, offer a glimpse into the polycrystalline morphology within the sample area. The corresponding energy-dispersive X-ray spectroscopy analysis in Figure 4b proved the existence of In in the SnTe matrix. Figure 4c provides a visual representation of the impact of high-pressure treatment on the sample morphology. It illustrates that the high-pressure synthesis leads to samples enriched with well-defined nanograins. These nanograins are known to enhance phonon scattering, which is crucial for reducing the thermal conductivity of the material. Inverse fast Fourier transform (IFFT) images, as shown in Figure 4d,e, reveal notable lattice distortions and dislocations within the structure. These lattice distortions and dislocations are a direct result of the high-pressure synthesis process and are instrumental in scattering both charge carriers and short-wavelength phonons. The presence of In_2_Te_3_ nanoregions within the high-pressure-synthesized samples, as identified in Figure 4c, further contributes to the scattering mechanisms. The combined effects of nanograins, secondary nanophases, and the high density of dislocations are responsible for the scattering of charge carriers and phonons. This scattering results in a decrease in *μ*_H_ and *κ*_ph_, which are both beneficial for enhancing the thermoelectric performance of the material. Notably, the mean free path of phonons typically exceeds that of electrons, suggesting that phonon scattering is more pronounced. The microstructural analysis presented in Figure 4 provides a comprehensive understanding of the factors that influence the thermoelectric properties of the synthesized samples. These insights are crucial for guiding future material design and synthesis strategies aimed at optimizing thermoelectric performance.

**Figure 5:**
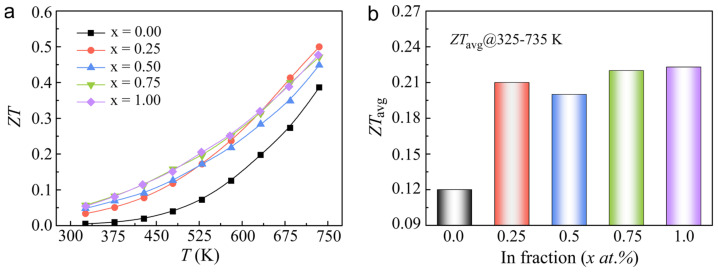
Temperature dependence of (**a**) figure of merit (*ZT*); (**b**) comparision for *ZT*_ave_ values of for In*_x_*Sn_1−*x*_Te (*x* = 0, 0.25 at.%, 0.5 at.%, 0.75 at.%, 1 at.%) at the temperature range of 325–735 K.

Figure 5a presents a comprehensive overview of the temperature-dependent figure of merit (*ZT*) for the In*_x_*Sn_1−*x*_Te samples with varying In concentrations (*x* = 0, 0.25 at.%, 0.5 at.%, 0.75 at.%, and 1 at.%). The figure illustrates a clear trend of enhanced *ZT* values across the entire temperature range, attributed to the combined effects of an increased Seebeck coefficient and decreased thermal conductivity. Notably, the sample with *x* = 0.25 at.% In achieves a maximum *ZT* of approximately 0.5 at 735 K, representing a significant enhancement of nearly 30% compared to the undoped SnTe. This peak *ZT* value is a testament to the effectiveness of In doping in improving the thermoelectric performance of SnTe. Beyond the peak *ZT* value, the average *ZT* (*ZT*_avg_) across a broad temperature range serves as a critical metric for evaluating the practical feasibility of thermoelectric devices. The *ZT*_avg_ provides a more representative measure of the material’s thermoelectric performance over a range of operating temperatures, which is crucial for practical applications. The In_0.0075_Sn_0.9925_Te and In_0.01_Sn_0.99_Te sample exhibits the highest *ZT*_avg_ of approximately 0.22 over the temperature interval of 325–735 K, signifying an impressive 83% improvement relative to the undoped SnTe. This substantial enhancement underscores the promising potential of In-doped SnTe for high-efficiency thermoelectric applications. The results presented in Figure 5a,b highlight the significant advancements achieved through the In doping and high-pressure synthesis of SnTe. In conclusion, the microstructural and thermoelectric analyses presented in this study demonstrate the effectiveness of In doping and high-pressure synthesis in enhancing the thermoelectric properties of SnTe. These advancements pave the way for the development of high-performance thermoelectric materials that could play a significant role in sustainable energy technologies.

## 3. Experimental Section

Samples of In*_x_*Sn_1−*x*_Te (*x* = 0, 0.25, 0.5, 0.75, 1.0 at.%) were synthesized using high-purity indium (In, powder, 99.99%), tin (Sn, powder, 99.99%), and tellurium (Te, ingot, 99.999%) as starting materials, adhering to the specified stoichiometric ratios. The precursor mixtures were ground for 30 min at ambient temperature in the glove box and then transferred into quartz tubes. These tubes were sealed under vacuum (10^−4^ Torr) and subsequently heated to 923 K, where they were held for 40 h. The samples were then sintered at 1173 K for 10 h and cooled to room temperature naturally. The ingots were crushed, shaped, and fabricated into cylinders with a diameter of approximately 12 mm and a thickness of about 5 mm for high-pressure and high-temperature (HPHT) experiments. These experiments were carried out using a sophisticated high-pressure apparatus (ZN-460, China), which provided the conditions of 4 GPa and 1473 K. Each HPHT run lasted approximately 30 min, which was sufficient for the sample molding under extreme conditions. After the HPHT treatment, the cylindrical ingots were removed from the apparatus and cut and polished into bars and disks. The bars were intended for subsequent measurements of electrical transport properties, while the disks were prepared for the evaluation of thermal properties.

After HPHT synthesis, the phase structure of all samples was characterized using X-ray diffraction (XRD) with Cu-K*α* radiation (λ = 1.5406 Å, Rigaku SmartLabSE, Japan). The XRD analysis provided crucial information about the presence of any secondary phases, lattice parameters, and the overall phase purity of the samples, which are all vital for understanding the thermoelectric performance. The microstructural morphology of the bulk samples was examined via scanning electron microscopy (SEM, Carl Zeiss Sigma 500 VP, Carl Zeiss, Oberkochen, Germany) equipped with energy-dispersive spectroscopy (EDS), and the transmission electron microscopy (TEM) images were obtained on a JEM-2100plus (Japan Electron Optics Laboratory, Tokyo, Japan) at an acceleration voltage of 200 kV, which provided high-resolution images that revealed the sample’s grain size, grain boundaries, and porosity. These morphological features are directly related to the material’s thermal and electrical transport properties. Hall effect measurements were performed using the van der Pauw method with a Hall measurement setup (Lake Shore 8400, Lake Shore, Columbus, OH, USA), which is capable of providing accurate measurements of the Hall coefficient, carrier concentration, and mobility. These measurements were crucial for understanding the electronic behavior of the doped SnTe samples and for assessing the effectiveness of the doping strategy. The electrical resistivity (*ρ*) and Seebeck coefficient (*S*) were measured concurrently using a CTA-3s apparatus (Cryoall, Peking, China) over a temperature range of 325 K to 735 K, with a temperature step of 50 K and a heating rate of 5 K min^−1^. The thermal conductivity (*κ*) was calculated using the equation *κ* = *DC*_P_*ρ*, where *D* is the thermal diffusivity coefficient obtained via the laser flash method using a Netzsch LFA457 instrument, *ρ* is the pellet density measured by the Archimedes method, and *C*_P_ is the specific heat capacity estimated using the Dulong–Petit law, assuming it to be temperature-independent. Both of these are fundamental for calculating the power factor and, ultimately, the figure of merit (*ZT*) of the thermoelectric materials. The uncertainties in *κ* and *ρ* are ±5–7%, while the uncertainty in *S* is ±5%.

## 4. Conclusions

In summary, this study comprehensively investigates the impact of In doping on the thermoelectric properties of SnTe under high pressure. We have successfully fabricated high-quality polycrystalline In*_x_*Sn_1−*x*_Te (*x* = 0, 0.25 at.%, 0.5 at.%, 0.75 at.%, 1 at.%) samples through an HPHT method. These results indicate that the incorporation of In significantly improves the power factor of SnTe. The In_0.0075_Sn_0.9925_Te composition exhibits power factor enhancements of approximately 6.5 times at 325 K compared to the pristine SnTe. This improvement is attributed to the increase in the Seebeck coefficient. In addition, the thermal conductivity of In_0.0025_Sn_0.9975_Te samples decreased by about 33% at 325 K and 17% at 735 K. Furthermore, we observe an overall improvement in the *ZT* for SnTe across a broad temperature range from 325 K to 735 K, with the In_0.01_Sn_0.99_Te sample achieving a *ZT*_avg_ of 0.22. The results presented in this study contribute to a deeper understanding of the fundamental mechanisms governing thermoelectric performance and provide a roadmap for the optimization of SnTe-based materials for practical thermoelectric applications.

## Figures and Tables

**Table 1:** Hall coefficient (*R*_H_), carrier concentration (*n*_H_), and Hall mobility (*μ*_H_) of In*_x_*Sn_1−*x*_Te synthesized by high temperature and high pressure.

Content of In *x* (at.%)	Hall Coefficient *R_H_* (cm^3^ C^−1^)	Carrier Concentration *n* (cm^−3^)	Hall Mobility *μ* (cm^2^ V^−1^ s^−1^)
0.00	0.09	6.96 × 10^19^	933
0.25	0.11	5.72 × 10^19^	490
0.50	0.07	8.97 × 10^19^	212
0.75	0.06	1.00 × 10^20^	154
1.00	0.06	9.74 × 10^19^	124

## Data Availability

The raw data supporting the conclusions of this article will be made available by the authors on request.
